# Physical activity of children and adolescents with Charcot-Marie-Tooth neuropathies: A cross-sectional case-controlled study

**DOI:** 10.1371/journal.pone.0209628

**Published:** 2019-06-12

**Authors:** Rachel A. Kennedy, Kate Carroll, Kade L. Paterson, Monique M. Ryan, Joshua Burns, Kristy Rose, Jennifer L. McGinley

**Affiliations:** 1 Department of Neurology, The Royal Children’s Hospital, Parkville, Victoria, Australia; 2 Murdoch Children’s Research Institute, Parkville, Victoria Australia; 3 Department of Physiotherapy, The University of Melbourne, Parkville, Victoria, Australia; 4 Centre for Health, Exercise and Sports Medicine, Department of Physiotherapy, The University of Melbourne, Parkville, Victoria, Australia; 5 Department of Paediatrics, The University of Melbourne, Parkville, Victoria, Australia; 6 The University of Sydney & The Children’s Hospital at Westmead, Sydney, New South Wales, Australia; Providence Care Hospital, CANADA

## Abstract

**Background:**

Disability related to the progressive and degenerative neuropathies known collectively as Charcot-Marie-Tooth disease (CMT) affects gait and function, increasing with age and influencing physical activity in adults with CMT. The relationship between CMT-related disability, ambulatory function and physical activity in children and adolescents with CMT is unknown.

**Method:**

A cross-sectional case-controlled study of physical activity in 50 children with CMT and age- and gender-matched typically developing (TD) controls [mean age 12.5 (SD 3.9) years]. A 7-day recall questionnaire assessed physical activity; CMT-related disability and gait-related function were measured to explore factors associated with physical activity.

**Results:**

Children with CMT were less active than TD controls (estimated weekly moderate to vigorous physical activity CMT 283.6 (SD 211.6) minutes, TD 315.8 (SD 204.0) minutes; *p* < 0.001). The children with CMT had moderate disability [CMT Pediatric Scale mean score 17 (SD 8) /44] and reduced ambulatory capacity in a six-minute walk test [CMT 507.7 (SD 137.3) metres, TD 643.3 (74.6) metres; *p* < 0.001]. Physical activity correlated with greater disability (ρ = -0.56, *p* < 0.001) and normalised six-minute walk distance (ρ = 0.74, *p* < 0.001).

**Conclusions:**

CMT-related disability affects physical activity and gait-related function in children and adolescents with CMT compared to TD peers. Reduced physical activity adversely affects function across the timespan of childhood and adolescence into adulthood in people with CMT.

## Introduction

Physical activities involving walking, running and jumping are often limited by progressive muscle weakness in individuals affected by the inherited peripheral neuropathies collectively known as Charcot-Marie-Tooth disease (CMT) [1, 2]. Reduced physical activity is associated with negative health outcomes irrespective of disability, with a compounding effect in adulthood [3–5]. Children with physical disabilities are less physically active than their typically developing peers, with the potential for poorer health outcomes including obesity related to inactivity [6, 7]. Likewise, for children and adolescents (“children”) with CMT, the negative health consequences of reduced physical activity are likely to compound CMT-related disability. However, to date there have been no studies of physical activity in children with CMT.

The natural history of CMT is of progressive weakness and worsening of disability through the lifespan. Adults with CMT are less physically active than typically developing adults [8]. Furthermore, adults with a greater degree of disability, report being less active than adults with milder disability [9]. Whilst adults with CMT are more likely to experience greater disability-related limitations than children with CMT, weakness in CMT is evident from early childhood [10] and disability increases throughout childhood and adolescence [11]. Therefore, it is possible that children with CMT are less active than their typically developing peers due to CMT-related disability and are at risk of negative health outcomes related to inactivity.

Understanding physical activity in children’s daily lives requires a measurement tool that reflects activity throughout the day. There is no single method of measuring physical activity in children that captures both objective and descriptive information about the type of activity undertaken [12]. A review of physical activity monitoring outlined several paediatric-specific self-report physical activity tools that can be completed within a single study visit and have been validated with objective measures of activity (accelerometers and activity monitors) [13]. Self-report recall questionnaires record activities undertaken at school and out of school hours, during organised extra-curricular and week-end activities, and incidental activities such as active mobility in the community [13].

Disability in children with CMT likely affects engagement in recreational and organised sports, as well as incidental activities such as using stairs and walking longer distances. Gait-related activities undertaken safely and sufficiently in the everyday environments of daily life can be defined as functional ambulation [14]. It is likely that children with CMT are less physically active, and also report and demonstrate limitations to functional ambulation. Therefore, the aims of this study were to compare self-reported physical activity of children and adolescents with CMT to that of age- and gender-matched typically developing (TD) peers; and secondly, to investigate associations between physical activity, CMT-related disability and measures of functional ambulation in children and adolescents with CMT.

## Methods

### Study design

A cross-sectional, case-controlled study was conducted across two specialist paediatric neuromuscular centres. Ethical approval was gained from The Royal Children’s Hospital (RCH) (HREC 33272 and 36019), The Sydney Children’s Hospitals Network (LNR/16/SCHN/132) and The University of Melbourne (1647763). Parents/guardians and participants 12 years and older were provided with a plain language information statement, and participant assent and parent/guardian written consent were gained prior to enrolment.

### Participants

Fifty ambulant children with a confirmed genetic or clinical diagnosis of CMT were recruited from either the Neuromuscular Clinic at The Royal Children’s Hospital (RCH), Melbourne, or the Peripheral Neuropathy Management Clinic at Children’s Hospital, Westmead (CHW), Sydney between January 2016 and October 2017. Fifty age- and gender-matched typically developing (TD) children who did not have CMT and resided in Melbourne and regional Victoria were recruited from families known to the researchers and unaffected siblings of the participants with CMT. For all participants (CMT and TD), inclusion criteria included the ability to walk independently > 75 metres (orthotics permitted); exclusion criteria included a history of musculoskeletal, developmental or other neuromuscular conditions that could affect gait, and lower limb surgery or injury in the preceding 6 months that might limit physical activity or gait.

### Procedures and assessments

A single clinical assessment was conducted in the respective outpatient clinic by a clinician trained in the use of the outcome measures [15].

#### Participant descriptors and characteristics

Participant descriptors and characteristics including date of birth, standing height and weight, dominant hand and foot, and CMT subtype (CMT only) were recorded and body mass index (BMI) calculated (kg/m^2^). Disability in the participants with CMT was assessed with the Charcot-Marie-Tooth disease Pediatric Scale (CMTPedS) a composite 11-item, well-validated and reliable, linearly-weighted outcome measure of disability in children and adolescents aged 3–20 years which measures motor function, strength, and balance [16]. The TD participants were assessed on selected items from the CMTPedS relating to lower limb function; specifically, dorsiflexor and plantar flexor strength, standing balance, lower limb power with a standing long jump and the six-minute walk test (6MWT). Additional foot and ankle characteristics assessed in all participants included foot posture index (FPI) [17].

#### Physical activity questionnaire–child (PAQ-C)

The PAQ -C is a valid and reliable self-reported, 7-day recall measure of physical activity in children [18–20]. The PAQ-C was used for all participants enrolled in this study irrespective of age, as it aligns with the typical daily activity schedule within the Australian school system [19]. Participants were asked to report the types and intensity of physical activity in the seven days prior to their study visit. If the visit was undertaken during school holidays, the child was asked to reflect on the last seven days that they were at school, as extra-curricular activities are often suspended over holiday periods, altering physical activity patterns. Children aged 8 years and younger completed the PAQ-C with assistance of a parent or adult.

The PAQ-C consists of nine questions scored on a 5-point Likert scale (S1 Appendix) [19]. The first relates to a checklist of physical activities, both sporting and leisure, common to children and adolescents. The participant was asked to rate how often they performed these activities in the past seven days on a 5-point scale from “none” to “7 or more”. Minor modifications were made to the checklist for this study to reflect activities common to Australia, in keeping with prior reports from other cultures [21–23]. For example, activities related to snow sports were removed and Australian sports such as cricket and netball were added (see S1 Appendix for full list of activities). The remaining eight questions utilised a 5-point ordinal scale to assess the level of physical activity (none through to vigorous); during the school day (PE/sports class, recess and lunch breaks), immediately after school, in the evenings and on the week-end; a general statement relating to how physically active the child was during the past 7 days; and a general statement relating to physical activity levels for each day of the previous week.

The PAQ-C is scored out of five, with 1/5 reflecting low activity and 5/5 reflecting high activity [24]. From the PAQ-C score, a group estimate of moderate to vigorous physical activity (MVPA) was derived from a method calibrated against accelerometry [25]. This method was used to calculate a group estimate of weekly MVPA for the CMT and TD groups.

#### Assessments of functional ambulation

The 6MWT was utilised as a test of ambulatory capacity and was conducted according to the local clinic’s usual practice and following the CMTPedS protocol [16]. In Melbourne, participants wore their own well-fitting footwear (typically a pair of athletic-type runners or similar), with the addition of orthotics if usually worn. The course was a 20-metre-long circuit on a flat vinyl surfaced corridor in a hospital outpatient clinic. In Sydney, participants were assessed barefoot unless they required ankle foot orthoses (AFOs), in which case they wore AFOs with their usual footwear. The course was a 25-metre-long circuit on a flat carpeted surface in a hospital outpatient clinic. Six-minute walk distance (6MWD) was normalised to height to account for differences in height across the large age range of participants [26].

For the participants with CMT, a further questionnaire was completed to assess the effect of CMT-related impairments on gait function. The Walk-12 is a validated self-reported questionnaire which examines the effect of CMT on gait and gait-related activities [27]. Briefly, 12 statements relating to gait and gait-related activities were answered on a 5-point Likert scale from zero (0) “my neuropathy does not affect this” to five (5) “I am very limited by my neuropathy”. The raw score out of 60 was transformed to provide a composite score; a higher score indicating greater perceived CMT-related limitations to gait [28]. Participants with CMT aged 8 years and younger were able to be assisted by a parent or adult to complete the questionnaire.

### Data management

Data from the clinical assessments were recorded on paper case report forms (CRF) and subsequently entered in an electronic data management system (REDCap), hosted by the Murdoch Children’s Research Institute [29]. Data from the two questionnaires (PAQ-C and Walk-12) were either completed on a paper CRF or entered directly into REDCap by the participants during the study visit. Paper CRFs from Sydney were scanned and emailed to Melbourne for data entry.

### Sample size calculation and statistical analysis

Assuming normality of the PAQ-C data, a sample size of 50 in each group with α_1_ = 0.05 and a moderate effect size (d = 0.50) allowed for >80% power to detect differences between the groups. When considering the power of the correlation co-efficient analysis a sample of 50 with r = 0.5, α_1_ = 0.05 allowed for >90% power to detect associations between the CMTPedS, Walk-12 and estimated weekly MVPA.

Normality of data distribution was visually examined and statistically tested for all variables with Shapiro-Wilk test. Appropriate descriptive statistics were used to characterise the data. Based on the type and distribution of the data, parametric (*t*-test) or non-parametric (Wilcoxon signed-ranks test) tests were used to assess for differences between the CMT and TD groups. Effect size was calculated with Cohen’s *d*. A linear regression determined the effect of CMT status (CMT or TD), age, height and site on estimated weekly MVPA. Spearman’s correlations were used to determine associations between physical activity (estimated weekly MVPA), and CMT-related disability (CMTPedS), ambulatory capacity (normalised 6MWD) and perceived walking ability (Walk-12). The independent variable with the strongest relationship to physical activity was entered into a linear regression with selected covariates to further explore this relationship. An α level was set at 0.05 for statistical significance. Stata v15 was used for data analysis.

## Results

### Participants

One hundred children, 50 with CMT and 50 age- and gender-matched TD controls, participated in this study with a mean age of 12.5 years (range 4–18 years old). CMT subtypes included CMT1A (n = 37), CMTX (n = 6), CMT2 (n = 2), CMT1B (n = 1), CMT4A (n = 1), CMTDIB (n = 1) and unknown/no genetic diagnosis (n = 2). Participant characteristics are outlined in Table 1. There were no statistical differences in height and weight between the groups. The CMT group had a higher BMI compared to the TD group (*p* = 0.003) but remained within a healthy range of 16–21 kg/m^2^ for the mean age of the study groups [30]. The participants with CMT were moderately disabled, had weaker ankle dorsiflexor muscles, poorer balance and reduced lower limb muscle power as assessed with a standing long jump (all *p* < 0.001) compared to their TD peers. The group of participants with CMT recruited from Sydney were older (CHW 14.4 years [SD 2.5], RCH 11.2 years [SD 4.1]; *p* = 0.003) however there was no difference in CMT-related disability between the two groups of CMT participants (CMTPedS CHW 20/44 [SD 10], RCH 16/44 [SD 7]; *p* = 0.11).

**Table 1:** Participants’ characteristics and clinical measures (CMT and TD).

	CMT	TD	Mean difference	Effect size *d*	*p* value
	Mean (SD) [95% CI]	Mean (SD) [95% CI]	Mean (SD) [95% CI]		
**Gender male: female**	24:26	24:26	-	-	-
**Age (years)**	12.5 (3.9) [11.4, 13.6]	12.5 (3.9) [11.4, 13.6]	-	-	-
**Height (m)**	1.51 (0.20) [1.46, 1.57]	1.51 (0.20) [1.46, 1.57]	-	-	0.9
**Weight (kg)**	49.1 (21.8) [43.0, 55.3]	44.5 (17.2) [39.6, 49.4]	4.7 (17.9) [0.4, -9.8]	-	0.07
**BMI (kg/m^2^) ^1^**	20.5 (5.9) [18.8, 22.2]	18.6 (3.5) [17.5, 19.6]	2.0 (5.7) [0.4, 3.6]	-	0.003
**FPI—left median (IQR) (-12 to +12) ^1^**	0 (-2 to 6)	5 (3 to 6)	5	-	< 0.001
**FPI—right median (IQR) (-12 to +12) ^1^**	1 (-2 to 6)	5 (2 to 6)	4	-	0.01
**Dorsiflexor strength (Newtons) ^a^**	47.3 (27.4) [39.5, 55.1]	90.1 (25.6) [82.8, 97.4]	42.8 (37.6) [39.5, 55.1]	1.6	< 0.001
**Plantar flexor strength (Newtons) ^a^**	146.0 (76.5) [124.2, 167.7]	153.2 (43.7) [140.8, 165.6]	7.2 (82.2) [-16.1, 30.6]	0.12	0.5
**CMTPedS (/44)**	17 (8) [15, 20]	-	-	-	-
**Balance BOT (/37) ^a^**	19 (9) [17, 22]	32 (3) [31, 33]	12.1 (9.2) [9.5, 14.7]	1.8	< 0.001
**Standing long jump (m) ^a^**	0.69 (0.38) [0.58, 0.80]	1.42 (0.32) [1.33, 1.51]	0.73 (0.50) [0.59, 0.87]	2.0	< 0.001
**6MWD (m) ^a^**	507.7 (137.3) [468.7, 546.7]	643.3 (75.6) [621.9, 664.8]	-135.6 (148.4) [-177.8, -93.4}	1.2	< 0.001
**N6MWD ^b^**	341.9 (95.7) [314.7, 368.1]	429.4 (56.0) [413.5, 445.3]	-87.5 (87.9) [-112.5, -62.6]	1.1	< 0.001

^1^ Wilcoxon signed-ranks test;

^a^ selected lower limb items from the CMTPedS completed by TD participants;

^b^ 6MWD normalised to height. Abbreviations: 6MWD: six-minute walk distance; BMI: body mass index; BOT: Bruininks-Oseretsky test of motor proficiency; CMT: Charcot-Marie-Tooth disease; CMTPedS: CMT Pediatric scale; ES: effect size; FPI: Foot posture index; IQR: interquartile rank; N6MWD: Normalised 6MWD to height; TD: typically developing

### Measure of physical activity

Participants with CMT were less physically active than their TD peers as reported on the PAQ-C (CMT median 2.2 [IQR 1.5, 2.7], TD median 2.8 [IQR 2.3, 3.3]; *p* < 0.001). The CMT reported group estimate of MVPA was 40 minutes per day (estimated MVPA 283.6 minutes per week [SD 211.6] 95% CI 222.8, 344.3), whereas the TD reported group estimate was 45 minutes per day (estimated MVPA 315.8 minutes per week [SD 204.0] 95% CI 257.2, 374.4) (*p* < 0.001). A linear regression with estimated weekly MVPA as the dependent variable, CMT status (CMT or TD) as the independent variable and height, age and site (RCH or CHW) as the covariates found that 95% of the variance in MVPA could be explained by CMT status, height and age (F (4, 94) = 404.69, R^2^ = 0.95, *p* < 0.001). Whether the children with CMT were from RCH or CHW did not influence their level of physical activity (Table 2). One participant with CMT did not complete the PAQ-C questionnaire.

**Table 2:** Relationship between physical activity (estimated weekly MVPA) and CMT status adjusted for height, age and site.

DV Estimated weekly MVPA	Coefficients β (B: 95% CI), *p* value
IV CMT status (CMT or TD)	-0.06 (-26.08: -49.26, -2.90), *p* = 0.028
Covariates	
Height	0.20 (2.11: 1.11, 3.12), *p* < 0.001
Age	-1.13 (-60.22: -65.33, -55.11), *p* < 0.001
Site (RCH or CHW)	-0.02 (-11.42: -41.23, 18.40), *p* = 0.449

DV: dependent variable; IV: independent variable; β: standardised regression coefficient; B: unstandardized regression coefficient; MVPA: moderate to vigorous physical activity; CMT: Charcot-Marie-Tooth disease; TD: typically developing; RCH: The Royal Children’s Hospital, Melbourne; CHW: Children’s Hospital Westmead, Sydney

### Measures of functional ambulation

Thirty-two participants with CMT completed the 6MWT in footwear and 18 walked barefoot due to site dependent protocol differences. Seven participants with CMT wore AFOs and two wore customised foot orthoses. All TD participants wore footwear without the addition of orthoses. Although footwear is known to influence gait speed and step length [1], there was no difference in 6MWD between the participants with CMT who wore shoes and those who walked barefoot (6MWD mean [SD] shoes 485 m [161], barefoot 548 m [66], *p* = 0.12).

Participants with CMT walked significantly shorter distances in six minutes compared to their TD peers with a large effect size (mean difference -135.6 m, *p* < 0.001, *d* = 1.2 (95% CI [-177.8, -93.4]); Table 1). When 6MWD was normalised to the participant’s height to account for the influence on step length, the significant difference remained, as did the large effect size (mean difference -87.5, *p* < 0.001, *d* = 1.1 (95% CI [-112.5, -62.6]); Table 1).

**Fig 1:**
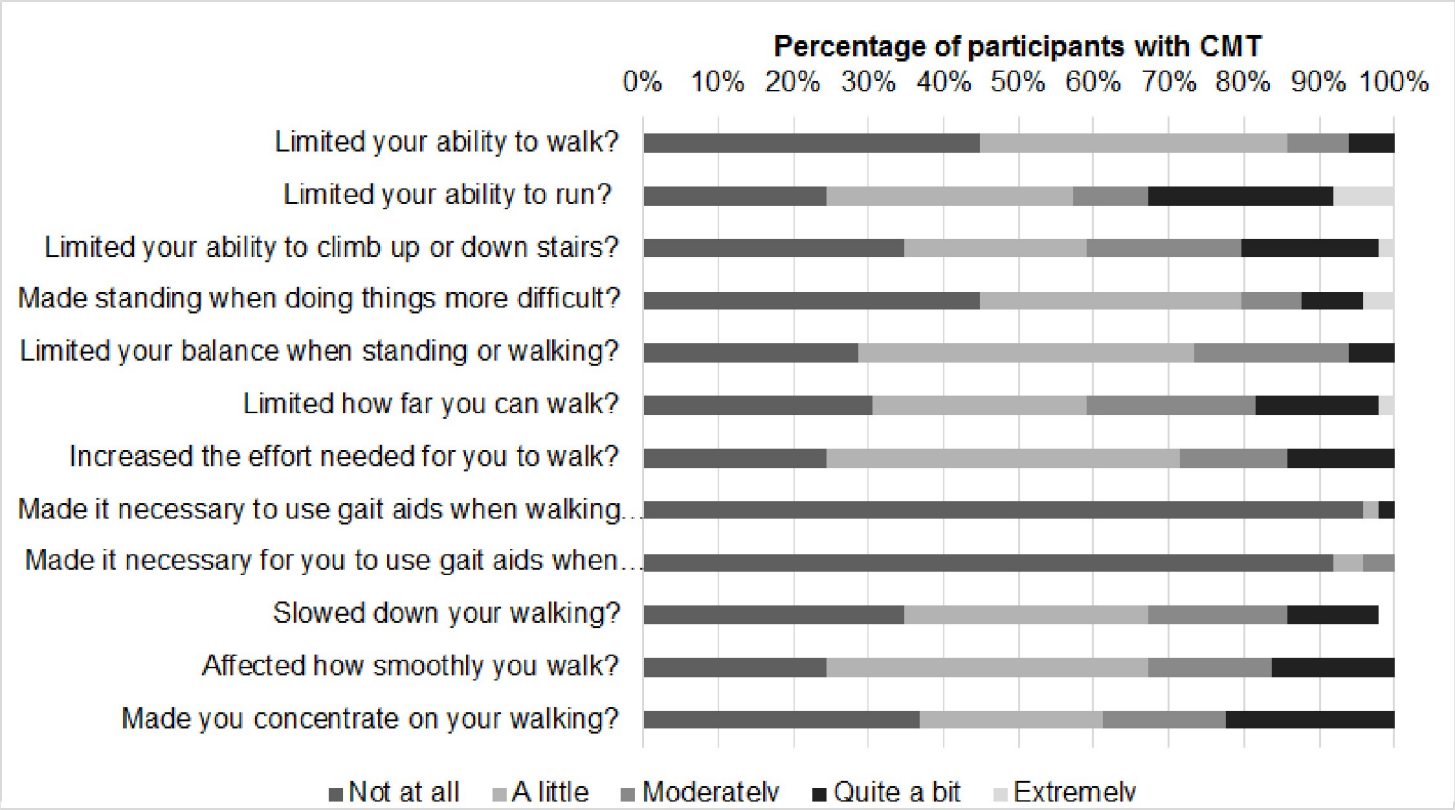
Percentage of participants with CMT (n = 49) responses to the question “How much has your neuropathy…”.

Participants with CMT reported a mild effect of CMT-related disability on gait and gait-related activities with a transformed Walk-12 mean score of 24.7% (mean 24.7 (SD 19) 95% CI [19.3, 30.2]). Closer inspection of the individual questions revealed that except for the use of gait aids, 50–75% of the participants felt that their CMT affected their gait and gait-related activities at least “a little bit” (Fig 1). Nearly 30% or more of participants reported moderate or greater effects on their ability to run (43%), ascend or descend stairs (41%), how far they could walk (41%), increased concentration required when walking (39%), the smoothness or co-ordination of their walking (33%), increased effort to walk (31%) and how fast they could walk (31%) (Fig 1). One participant with CMT did not complete the Walk-12 questionnaire.

### Associations between physical activity, disability, functional ambulation and perceived effect on gait and gait-related activities in participants with CMT

A Spearman’s correlation was calculated between physical activity (estimated weekly MVPA), CMT-related disability (CMTPedS), functional ambulatory capacity (normalised 6MWD) and perceived effect on gait and gait-related activities (Walk-12) in 49 participants with CMT. The strongest association was found between physical activity and functional ambulatory capacity (normalised 6MWD) (ρ = 0.74, *p* < 0.001) and a moderate inverse association was found between physical activity and CMT-related disability (CMTPedS) (ρ = -0.56, *p* < 0.001). There was a weak association between physical activity and self-reported perception of walking ability (Walk-12) (ρ = -0.34, *p* = 0.02). To gain a better understanding of the relationship between physical activity and functional ambulatory capacity, a linear regression was conducted with age, gender and site (RCH or CHW) entered as covariates. For the children with CMT, 98% of the variance in physical activity could be explained by functional ambulatory capacity, age and gender but not site (F (4, 44) = 486.32, *p* < 0.001, R^2^ = 0.98 (Table 3).

**Table 3:** Linear regression with physical activity as the dependent variable, functional ambulatory capacity the independent variable and age, gender and site as covariates.

DV Estimated weekly MVPA	Coefficients β (B: 95% CI), *p* value
IV Normalised 6MWD	0.08 (0.175: 0.056, 0.295), *p* = 0.005
Covariates	
Age	-0.91 (-49.21: -52.14, -46.28), *p* < 0.001
Gender	-0.21 (-89.72: -109.16, -70.27), *p* < 0.001
Site (RCH or CHW)	-0.03 (-13.97: -35.81, 7.88), *p* = 0.204

Abbreviations: DV = dependent variable; IV = independent variable; β = standardised regression coefficient; B = unstandardized regression coefficient; MVPA = moderate to vigorous physical activity; 6MWD = six-minute walk distance, normalised to height; RCH = The Royal Children’s Hospital, Melbourne; CHW = Children’s Hospital Westmead, Sydney

## Discussion

The findings of this study across two Australian paediatric neuromuscular centres suggest that children with CMT report being less physically active than their TD peers. Of note, nearly 75% of the children in this study were diagnosed with CMT1A, yet despite the high representation of this generally milder CMT sub-type, reported levels of physical activity and functional ambulation were considerably reduced [31]. Lower physical activity levels in the participants with CMT were associated with greater disability and reduced functional ambulation, both in terms of ambulatory capacity in a 6MWT and perceived disability-related limitations to gait and gait-related activities.

In this study we found that children with CMT are less active. It is therefore important in the context of a degenerative neuropathy with increasing impairments and limitations to monitor physical activity levels in children with CMT. Reduced physical activity is known to increase health-related illnesses in the general population and is likely to compound CMT-related disability. Maintaining general health and wellness is important for participation in education, paid employment and general activities of daily life. Understanding the importance of engaging and participating in physical activity whilst living with a physical disability is an important health message that health clinicians working with children with CMT should promote. It is important that the link between greater physical activity and better function is understood. The evidence from this study suggests that children with CMT who are more physically active, have less CMT-related disability and limitations, and greater capacity to undertake gait-related activities. Conversely, children with greater disability are less able to engage in physical activity leading to increased sedentary activity time. This may have a downward spiralling effect further reducing physical activity with greater impact on ambulatory function.

A strong relationship between physical activity and functional ambulatory capacity was found with children with CMT who reported being less physically active also demonstrating reduced ambulatory capacity. This has implications for community mobility where distance requirements in community settings can range up to 700 metres [32]. Children who are more disabled are further limited in their ability to access community activities that require traversing longer distances. The children with CMT who were less active had greater CMT-related disability and reported greater limitations to their gait-related activities, similar to the findings of a small study of children with mixed neuromuscular diagnoses [33]. Prior research suggests this may be a lifelong concern, as adults with CMT also report limitations to physical activity [8, 9]. The current study expands on the studies in adults to establish that physical activity is limited from childhood in CMT, similar to other paediatric disorders that present with physical disability-related limitations such as cerebral palsy, muscular dystrophy and spina bifida [7].

It is uncertain what intrinsic and extrinsic factors may facilitate or present as barriers to physical activity in children with CMT. The degeneration of the peripheral nerves in CMT causes lower limb weakness distally greater than proximally, leading to foot deformities, problems with balance, walking and frequent trips and falls [2]. Some evidence from children with other physical disabilities indicates that they may self-limit physical activity behaviours to avoid social embarrassment in sporting and social situations [34]. Similarly, it is likely that the risk of social embarrassment may also be a potential barrier to physical activity in children with CMT. Children with CMT are at a higher risk of falling [35]. The current study identified that children with CMT have impaired balance; over 60% considered that their walking was uncoordinated or less smooth and that they needed to concentrate on their walking. Children with CMT may self-limit physical activity to reduce and prevent falling or near-falls, similar to reports in young adults with cerebral palsy [36]. Further barriers may include difficulty accessing activities that are inclusive and provide opportunities to be physically active in educational and local community organisations [37]. Sourcing disability-trained and supportive trainers or coaches to adapt and modify activities for children with a physical disability is an ongoing problem in the Australian context [37].

There were several strengths to this novel study of physical activity in children with CMT. A well-described CMT cohort included a broad age and developmental range spanning childhood and adolescence and comprised CMT sub-types representative of the clinical populations from two specialist paediatric neuromuscular centres. Disability related to CMT was well-characterised with the CMTPedS, a meaningful and validated composite measure in paediatric CMT. Generalisability from this sample of convenience to other CMT populations is likely, given that the level of CMT-related disability in our CMT cohort was similar to reported levels in a large international cohort of 520 children [31]. The age- and gender-matching to TD controls strengthened the study and placed the findings within the context of typical development.

Utilisation of the calibration method of estimating MVPA suggested by Saint-Maurice et al. (2014) provided a meaningful quantification of physical activity however it was limited to a group estimate only. Further, limitations of the self-report PAQ-C is that it relies on recall memory and the child’s perception of what constitutes physical activity. Self-report recall physical activity questionnaires are therefore less accurate compared to objective measures [12]. The use of an activity monitor or accelerometer would have provided a more robust measure of MVPA cut-points and enabled further comparison to National Physical Activity Guidelines [38]. Additionally, the PAQ-C did not measure sedentary time. The substitution of the culturally relevant sports in the PAQ-C may also be a limitation as these were not specifically assessed for face or content validity. The clinical outcome measures were collected per local protocols. Differences in 6MWT circuit length were unlikely to affect group 6MWD, as the typically developing cohort were tested on the shorter circuit. Therefore, any error due to circuit length and a greater number of turns was likely to have underestimated the group difference [39]. Restricting the Walk-12 to the children with CMT limited the interpretation of these findings relative to what is typical for children without CMT.

Children with CMT are at risk of greater health problems and limitations due not only to their neuropathy but also disuse associated with reduced physical activity. Objective quantifiable assessment of physical activity in paediatric CMT, and investigation of potential barriers to physical activity including fear of falling, are areas that require further enquiry. Further investigation of enablers of physical activity is suggested, including the effects of exercise and increased physical activity on ambulatory function in children with CMT. Strength training improves activities of daily living in adults with CMT [40], and is safe in children with CMT [41]. In TD children, increasing physical activity from one 60-minute physical education class a week to five 40-minute classes a week improved muscle strength [42]. Given these findings, the effectiveness of age-appropriate regular exercise programs, utilising motivational behaviour change coaching and focussing on strength training and whole-body activity for children with CMT, requires further development and empirical investigation. Additionally, facilitating access to and participation in community-based recreational and sporting activities, including specialist training to enable coaches to adapt programs to include children with physical disabilities, is required.

## Conclusion

Physical activity and functional ambulation are adversely and significantly affected, and are associated with greater disability, in children with CMT. Healthcare clinicians, researchers and funding agencies ought to engage with and promote opportunities for children with CMT to be more physically active, be it through participation in structured, evidence-based exercise and training programs, or community-based recreational sporting programs. Further research is required to determine whether facilitating greater physical activity may slow degeneration and improve physical function in the everyday lives of children and adolescents with CMT.

## Supporting information

S1 Appendix Physical activity questionnaire. (PDF)

## Abbreviations

6MWT: six-minute walk test
6MWD: six-minute walk distance
AFOs: ankle foot orthoses
BMI: body mass index
CMT: Charcot-Marie-Tooth disease
CMTPedS: Charcot-Marie-Tooth Disease Pediatric Scale
CRF: case report form
MVPA: moderate to vigorous physical activity
N6MWD: normalised six-minute walk distance
PAQ-A/-C: physical activity questionnaire–adolescent/child
TD: typically developing
