# Supplementary material for: Physical activity of children and adolescents with Charcot-Marie-Tooth neuropathies: A cross-sectional case-controlled study
Source: PLoS One. 2019 Jun 12;14(6):e0209628. doi: 10.1371/journal.pone.0209628 (PMC6561632; doi:10.1371/journal.pone.0209628)
Supplement: S1 Appendix — (PDF) [file pone.0209628.s001.pdf]

Supplementary material – Physical Activity Questionnaire – child (PAQ-C) adapted for Australian participants

Instructions

- **We would like to know about your level of physical activity from the last 7 days (the last week).**
- **This includes sports or dance that make you sweat or make your legs feel tired, or games that make you breathe hard, like playing chasey, skipping, running, climbing and others.**
- **There are no right or wrong answers – this is not a test.**
- **Please answer all the questions as honestly and accurately as you can – this is important.**
- **If your parent or another adult helped you fill in this questionnaire please tick the box "I had help ☺".**
- **If you have any concerns about these questions or how they make you feel, please let your parent and / or the physiotherapist who is doing your assessment know.**

Study ID:	Date:	I had help ☺

1. Physical activity in your <u>spare time</u> (time not in school or in organised sports / classes): Have you done any of the following activities in the past 7 days (last one week)? If yes, how many times? Tick one box per row.

	No	1-2	3-4	5-6	7 times or more.
Skipping					
Water sports					
Roller blading/skating					
Chasey / tag					
Walking for exercise					
Walking to/from school or public transport					
Bicycling for fun / recreation					
Bicycling to/from school					
Jogging or running					
Aerobics / gym					
Swimming for fun/recreation					
Cricket / baseball / softball					
Dance					
Football					
Soccer					
Skateboarding					
Hockey					

Basketball / netball					
Other					

2. In the last 7 days, during your sports and / or physical education (PE) classes, how often were you very active (playing hard, running, jumping, throwing)? (Tick one only)

I don't do PE	
Hardly ever	
Sometimes	
Quite often	
Always	

3. In the last 7 days, what did you do most of the time *at recess / little play*? (Tick one only)

Sat down (talking, reading, doing schoolwork)	
Stood around or walked around	
Ran or played a little bit	
Ran around and played quite a bit	
Ran and played hard most of the time	

4. In the last 7 days, what did you normally do at lunch (besides eating lunch)? (Tick one only)

Sat down (talking, reading, doing schoolwork)	
Stood around or walked around	
Ran or played a little bit	
Ran around and played quite a bit	
Ran and played hard most of the time	

5. In the last 7 days, on how many days right after school did you do sports, dance or play games in which you were very active? This can include Out of school hours care (OHSC) (Tick one only)

None	
1 time last week	
2 or 3 times last week	

4 times last week	
5 times last week	

6. In the last 7 days, on how many <u>evenings</u> did you do sports, dance or play games in which you were very active? (Tick one only)

None	
1 time last week	
2 or 3 times last week	
4-5 times last week	
6-7 times last week	

7. *On the last week-end*, how many times did you do sports, dance or play games in which you were very active? (Tick one only)

None	
1 time	
2 or 3 times	
4-5times	
6 or more times	

8. Which *one* of the following describes you best for the last 7 days? Read *all five* statements before deciding on the *one* answer that describes you. (Tick one only)
   A. All or most of my free time was spend doing things that involve little physical effort
   B. I sometimes (1-2 times last week) did physical things in my free time (eg. Played sports, went running, swimming, bike riding etc)
   C. I often (3-4 times last week) did physical things in my free time
   D. I quite often (5-6 times last week) did physical things in my free time
   E. I very often (7 or more times last week) did physical things in my free time

9. Mark how often you did physical activity (like playing sports, games, dancing or any other physical activity) for each day last week. (Tick one box per row)

	None	A little bit	Medium	Often	Very often
Monday					
Tuesday					
Wednesday					
Thursday					
Friday					
Saturday					

Sunday					

10. Were you sick last week, or did anything prevent you from doing your normal physical activities? (Tick on only)

    Yes If yes, what prevented you? ___________________________________

    No

Adapted from Kowalski KC, Crocker PRE, Donen RM. The Physical Activity Questionnaire for Older Children (PAQ-C) and Adolescents (PAQ-A) Manual. Saskatoon, Canada: University of Saskatchewan; 2004.
